# Case Report: Histamine-mediated inflammation and mast cell modulation in central serous chorioretinopathy: a two case illustrations

**DOI:** 10.3389/fimmu.2026.1799560

**Published:** 2026-10-07

**Authors:** Sara KamaliZonouzi, Parsa Babaei Zadeh, Marko M. Popovic, Michael B. Gorin, Tal Eshkoly-Lior

**Affiliations:** 1 School of Medicine, Tehran University of Medical Sciences, Tehran, Iran; 2 Faculty of Medicine and Dentistry, University of Alberta, Edmonton, AB, Canada; 3 Department of Ophthalmology and Vision Sciences, University of Toronto, Toronto, ON, Canada; 4 Department of Ophthalmology, Unity Health Toronto, Toronto, ON, Canada; 5 Department of Ophthalmology and Human Genetics, University of California, Los Angeles, Los Angeles, CA, United States; 6 Retinal Disorders and Ophthalmic Genetics Division, Stein Eye Institute, University of California, Los Angeles, David Geffen School of Medicine at UCLA, Los Angeles, CA, United States

**Keywords:** central serous retinopathy, CSCR, histamine, mast cell, topical corticosteroid

## Abstract

**Introduction:**

Central serous chorioretinopathy (CSCR) is characterized by retinal pigment epithelium dysfunction and choroidal hyperpermeability. Beyond established associations such as psychosocial stress and corticosteroid exposure, experimental and epidemiological findings raise the possibility that mast cell and histamine-related pathways may contribute to CSCR pathophysiology. This study reviews mechanistic and clinical evidence for the role of histamine in the pathogenesis of CSCR, as well as the role of histamine-targeted therapies in this condition. We also present two cases, highlighting the possible therapeutic role of anti-allergy agents in CSCR.

**Methods:**

We searched Ovid MEDLINE, Embase, and Cochrane CENTRAL to October 2025 for studies of antihistamines and/or mast-cell stabilizers, alone or in combination in central serous retinopathy and pachychoroid spectrum diseases. Eligible designs included human observational and controlled studies. Two reviewers independently screened, extracted, and appraised data using ROBINS-I, NOS, and GRADE. Results were narratively synthesized.

**Results:**

After screening 316 articles, three studies (430 patients, 390 controls) from India and the United States were included. Direct evidence for anti-allergy therapeutics was limited to a single management series, in which short-course antihistamine treatment showed no meaningful visual or anatomical benefit; however, improvements occurred spontaneously or after focal laser. One case–control study reported a higher prevalence of antihistamine use and allergic disorders among CSCR patients (OR 4.1), suggesting a possible histamine-mediated risk. Another case-control study found no statistically significant association between antihistamine use and CSCR after multivariable adjustment.

**Conclusion:**

Analysis of the literature revealed inconsistent associations between antihistamine use and CSCR risk, with a brief therapeutic trial offering no benefit for acute CSCR. However, two illustrative cases demonstrate temporal associations between interruption of anti-allergic therapy and subsequent CSCR activity, followed by anatomical improvement after treatment re-initiation or modification. These observations are hypothesis-generating but cannot establish therapeutic efficacy or causality, particularly given concurrent treatment changes in case 1. Given the inconclusive therapeutic data, further randomized controlled trials are warranted to more rigorously evaluate sustained mast-cell stabilization regimens before drawing clinical conclusions.

## 1 Introduction

Central serous chorioretinopathy (CSCR, also known as central serous retinopathy) is part of the pachychoroid disease spectrum (PDS), which is defined by serous detachment of the neurosensory retina due to retinal pigment epithelium (RPE) dysfunction and choroidal hyperpermeability (1). It most often affects working-age adults and can cause metamorphopsia, reduced contrast sensitivity, and persistent or recurrent subretinal fluid that may lead to permanent visual loss (2). Risk factors include psychosocial stress, sympathetic overdrive, exposure to corticosteroids, and untreated hypertension (3). However, these associations do not fully explain the disease heterogeneity or variability in therapeutic efficacy.

Epidemiologically, CSCR predominantly affects men, with a male-to-female ratio of approximately 2.7:1, and the incidence peaks between 40 and 49 years of age at approximately 71.8 per 100,000 person-years. Male patients are diagnosed on average 3.3 years earlier than female patients. A recent systematic review and meta-analysis estimated that approximately 1.97 million individuals globally developed CSCR in 2025, with the highest absolute numbers occurring in Eastern and Southern Asia (4). Population-based studies in the United States have reported annual incidence rates of 9.9 per 100,000 in men and 1.7 per 100,000 in women, with a mean age at diagnosis of 41 years (5). Among associated comorbidities, allergic respiratory disease is particularly notable; a large Taiwanese population-based study of 2,921 CSCR patients found significant associations with allergic airway disease and corticosteroid use through multiple routes, including topical ophthalmic (OR 6.04), oral (OR 1.22), and nasal spray administration (6). These epidemiological findings underscore the overlap between CSCR and atopic/allergic conditions, suggesting shared immunological mechanisms.

Upon activation, mast cells release histamine, leukotrienes, proteases, and cytokines that can increase endothelial permeability, change vascular tone, and disrupt epithelial tight junctions. Preclinical work shows that targeted mast-cell degranulation in mice produces rapid dilation of choroidal vessels, increased choroidal thickness, serous retinal detachments, and retinal pigment epithelium tight-junction abnormalities with a concurrent rise in inflammatory cytokines. In that study, pretreatment with a mast cell stabilizer, disodium cromoglycate, reduced the rate of serous detachments (7). In a human study, administration of intravenous histamine increased subfoveal choroidal blood flow and dilation of retinal vessels (8, 9). However, this effect was attenuated by H1 receptor blockade and was not affected by H2 blockade, which implicates H1-mediated pathways in ocular vasodilation. Together, these data provide biologic plausibility for the role of histamine in pachychoroid conditions like CSCR, which may have therapeutic implications.

The mechanisms by which mast cells become activated in CSCR are likely multifactorial and extend beyond classical IgE-mediated degranulation. Mast cells express the high-affinity IgE receptor, and cross-linking of surface-bound IgE by allergens triggers immediate degranulation with release of preformed mediators including histamine, tryptase, and TNFβ (10). In patients with concomitant atopic disease, such as allergic rhinitis, asthma, or atopic dermatitis, elevated circulating IgE and persistent allergen exposure may tonically prime choroidal mast cells, lowering the threshold for degranulation. A case report of CSCR in a 14-year-old boy with severe atopic dermatitis supports this association, as disease onset coincided with topical corticosteroid treatment for his atopic condition (11). Beyond IgE-dependent pathways, complement fragments C3a and C5a generated through alternative pathway activation can directly stimulate mast cell degranulation via complement receptors, independent of immunoglobulin involvement. This complement-mediated route is particularly relevant given the established genetic association between complement factor H (CFH) variants and CSCR, suggesting that dysregulated complement activity in the choroid may constitute a non-allergic trigger for local mast cell activation (12). Additionally, neuropeptides, mechanical stress from choroidal venous congestion, and corticosteroid-induced changes in the choroidal milieu may each contribute to mast cell activation in susceptible individuals, independent of classical allergic pathways.

Clinical epidemiology also links CSCR to mast cell and histamine biology (13). CSCR often coexists with allergic disorders, such as allergic rhinitis and asthma, and with conditions marked by autonomic dysregulation or intermittent hypoxia such as obstructive sleep apnea (13, 14). These disease states can prime mast cells and alter choroidal hemodynamics. Overall, these findings provide biological plausibility for involvement of immune and histamine-related pathways in CSCR, but do not establish systemic immune dysregulation as a causal mechanism or demonstrate that antihistamines or mast-cell stabilizers are necessarily effective treatments.

Current treatment strategies primarily target symptoms rather than upstream causes of the RPE dysfunction (15, 16). Observation is used for acute, self-limited episodes, while chronic or vision-threatening disease may be managed with photodynamic therapy or focal laser when discrete leaks are present (17, 18). Pharmacologic approaches aimed at mineralocorticoid signaling and other pathways have shown mixed effectiveness, and there is no universally effective medical therapy that works for all CSCR cases (19–21). The randomized, double-blind, placebo-controlled VICI trial found no benefit of eplerenone over placebo for chronic CSCR (22). A recent multicenter retrospective case series from Mass Eye and Ear and UCSF reported anatomic improvements with faricimab, suggesting possible therapeutic benefits (23). Repurposing safe, widely available agents used in allergic disease has appeal in this context. H1 and H2 antihistamines, mast-cell stabilizers such as cromolyn sodium, and leukotriene-pathway inhibitors such as montelukast each target distinct paths in the mast-cell-histamine-leukotriene axis that influences choroidal permeability and RPE barrier function.

There remains a paucity of literature and understanding related to the role of histamine in CSCR pathogenesis and therapy. In this article, we synthesize mechanistic and clinical evidence that supports an immune contribution to CSCR, with an emphasis on mast cell activation and histamine signaling. Second, we review clinical and preclinical studies that evaluate antihistamines, mast-cell stabilizers, leukotriene-pathway inhibitors, and related anti-inflammatory strategies in CSCR and in PDS. Third, we present two illustrative cases that showcase the real-world potential of histamine-directed therapy, with attention to safety and adjunctive care. We aim to analyze the efficacy and safety of antihistamines and related agents in treating CSCR.

## 2 Materials and methods

### 2.1 Search strategy and eligibility criteria

We searched Ovid MEDLINE, Embase, and Cochrane CENTRAL from database inception through October 1, 2025. Core concepts combined condition terms (central serous retinopathy, central serous chorioretinopathy, CSR, CSCR) and the pachychoroid spectrum disorders (for which search terms included pachychoroid, pachychoroid pigment epitheliopathy, peripapillary pachychoroid syndrome (PPS), and polypoidal choroidal vasculopathy) with intervention or exposure terms. Intervention and exposure terms included antihistamines (H1 and H2) such as H1 agents (loratadine, cetirizine, fexofenadine, diphenhydramine, chlorpheniramine) and H2 agents (cimetidine, famotidine), as well as mast-cell stabilizers and dual-action antihistamine and mast-cell stabilizer agents across topical ocular or nasal, inhaled, and systemic routes: topical (cromolyn, olopatadine, ketotifen, bepotastine, epinastine, azelastine, lodoxamide), inhaled (cromolyn, nedocromil), and systemic (ketotifen, including oral formulations used outside the United States). To capture contextual evidence, we added keywords for risk factors and medication exposures. Reference lists of eligible studies and relevant reviews were hand searched, with targeted backward–forward citation searching performed to identify older management series and medication-associated reports. The study protocol was prospectively registered on September 19, 2025 with PROSPERO (CRD420251149548). (**Supplementary Table S1**).

We prespecified two evidence strata:

- Stratum A: Therapeutic studies. Peer-reviewed human studies reporting outcomes in adults with CSCR or PDS treated with an anti-allergic therapy (i.e. antihistamine and/or mast-cell stabilizers) of any class, dose, route, or schedule, either as monotherapy or with usual care. Eligible designs included randomized or nonrandomized comparative studies, prospective or retrospective observational cohorts, and case series.
- Stratum B: Contextual evidence. Peer-reviewed human studies that did not administer antihistamines as treatment but were relevant to the research question, including case–control studies assessing histamine or antihistamine exposure or related risk factors were considered. Single-patient case reports linking medication exposures or allergic conditions to CSCR with adequate ocular documentation were considered.

### 2.2 Exclusion criteria

Non-CSCR diagnoses without separable data, conference abstracts, editorials, reviews, and non-English publications were excluded.

### 2.3 Outcomes

For Stratum A, primary outcomes were best-corrected visual acuity and anatomic resolution of subretinal fluid on optical coherence tomography (OCT). Secondary outcomes included central retinal thickness, subfoveal choroidal thickness, time to fluid resolution, recurrence, need for rescue therapy, and adverse events. For Stratum B, outcomes included measures of association between exposures and CSCR and, when reported, visual recovery at prespecified time points.

### 2.4 Study selection and data collection

Titles and abstracts were screened by two independent reviewers (S.K.Z. and P.B.Z.). Full texts of potentially eligible records were assessed by two independent reviewers (S.K.Z. and P.B.Z.). Disagreements were resolved by consensus or, when needed, by a third independent reviewer (M.P.). Studies were classified into Stratum A or Stratum B prior to data extraction. (**Supplementary Table S2**).

For each included report, we extracted study design, setting, sample size, eligibility criteria, CSCR chronicity, imaging methods, intervention or exposure definitions, comparator, follow-up duration, outcome definitions and time points, adverse events, rescue therapy, and funding or conflicts.

### 2.5 Risk of bias and certainty of evidence assessment

Nonrandomized interventional studies were assessed using ROBINS-I. Case–control studies were appraised with the Newcastle–Ottawa Scale. Single-patient case reports were appraised with the JBI critical appraisal checklist for case reports. Two reviewers performed domain-level judgments independently and resolved discrepancies by consensus or third-reviewer adjudication. Certainty of evidence for key outcomes was graded using the GRADE approach for Stratum A; for Stratum B we summarized study-level limitations qualitatively.

### 2.6 Statistical analysis

Due to the scarcity of the available literature, no meta-analysis was performed. Results were synthesized narratively and summarized in structured tables. Visual acuity was abstracted in the format reported by each study, and no conversions to logMAR were undertaken. For the case–control studies, we extracted the authors’ reported measures of association, including odds ratios, confidence intervals, and P values, without re-calculation or pooling. For the interventional management series and the case reports, outcomes were described qualitatively based on the authors’ reported visual and imaging findings and timelines. No imputations or unit conversions were performed.

### 2.7 Case reports

We prospectively documented two illustrative cases that informed the review. For each case, we recorded demographic characteristics, relevant comorbidities, diagnostic criteria and chronicity, imaging protocols, anti-allergic therapy regimen and any adjunctive therapy, as well as OCT, visual outcomes, and adverse events over the follow-up duration. Patients provided written informed consent for publication. Identifiers were removed and presentation complied with local ethics policy.

## 3 Results

### 3.1 Study selection and characteristics

After screening 316 records, three studies with a total of 430 patients and 390 controls were included in the qualitative synthesis (**Table 1**). Two studies originated from India and one from the USA (24–26). The set comprised one single-center case series with prespecified clinic protocols (n=40 patients), one multicenter retrospective case-control study (312 cases and 312 controls), and one prospective matched case-control study that also examined visual recovery (78 cases and 78 controls) (24–26). Follow-up varied from 2–3 months to 1–2 years in cohort studies. Imaging confirmation of CSCR diagnosis with fluorescein angiography or OCT was reported in all studies. Acute, recurrent, and chronic phenotypes were represented.

**Table 1:** Study characteristics and results of included studies.

Study (first author, year; journal)	Country/setting	Design	N (patients/controls or eyes)	CSR phenotype/baseline	Exposure/intervention	Comparator	Outcomes measured	Follow-up	Main findings
Murthy, 1983; Indian J Ophthalmol (26)	India; single center	Management series (nonrandomized)	40 patients	Adults 30–44 years; mostly male; FA at baseline	Antihistamines for 4 days in 30; then vitamin C 500 mg daily ~2 months; 10 already on systemic steroids evaluated; laser per criteria if no improvement/worsening	None	BCVA; fundus; Amsler; FA; time to improvement; recurrence	Weekly first 2–3 months; recurrence tracked 1–2 years	Antihistamines showed no benefit in 29/30; 1 rapid improvement. 21/33 improved spontaneously within 5–10 weeks during placebo period; non-improvers improved after laser; recurrence in 6 over 1–2 years.
Haimovici, 2004; Ophthalmology (24)	USA; multicenter	Retrospective case–control	312 cases; 312 controls	Adults with CSCR vs matched controls	Self-reported exposures including antihistamines	Controls without CSCR	Odds of CSCR associated with exposures	Not applicable	Antihistamine use OR 3.9 (1.8–8.3) univariate; OR 2.5 (0.9–7.1), P = 0.0855 in stepwise model. Allergic respiratory disease OR 2.5 (1.2–5.1).
Das, 2020; Indian J Public Health Research & Development (25)	India; Regional Institute of Ophthalmology, Kolkata	Prospective observational case–control	78 CSCR cases; 78 controls	Acute, chronic, and recurrent CSCR; mean age 38.9 years; male:female 5.5:1; 61 first episode, 17 recurrent/chronic	Assessed systemic steroids, recent psychological stress/life events, smoking, alcohol, antihistaminic use; psychiatric evaluation; FFA as needed	Age- and sex-matched controls without CSCR	Associations of risk factors with CSCR; visual recovery at 1 year (BCVA ≥6/12 vs <6/12)	Every 2 weeks until resolution or up to 1 year	Significant associations: systemic steroids 14.1% vs 2.6% (P = 0.0173), stress 25.6% vs 12.8% (P = 0.0422), smoking OR 3.19 (P<0.05), antihistaminic use OR 4.104 (P = 0.0466). Alcohol not significant (OR 2.60, P = 0.4423). Visual recovery good in 89.7%; no risk factor significantly associated with poor recovery.

BCVA, best-corrected visual acuity; FA, fluorescein angiography; FFA, fundus fluorescein angiography; OCT, optical coherence tomography; SRF, subretinal fluid.

### 3.2 Anti-allergic therapies for management of CSCR

Direct therapeutic evidence was limited to a single management series in which 30 of 40 patients with CSCR received a 4-day antihistamine course at presentation (**Table 1**) (26). Twenty-nine of 30 showed no anatomical or visual benefit during that brief exposure, and one improved rapidly. During the subsequent placebo period with vitamin C, 21 of 33 improved spontaneously within 5 to 10 weeks. Patients without improvement or with worsening received focal laser per predefined criteria and improved thereafter. Six recurrences were documented over 1 to 2 years. No sustained antihistamine regimens and no dose-response evaluations were reported. No treatment-related adverse events were described.

### 3.3 Medication exposures and CSCR risk

Two studies assessed background medication exposures as opposed to directed treatment (see **Table 1**).

In the multicenter case-control analysis, self-reported antihistamine use was associated with risk of CSCR in univariable analysis (reported in 32/312 patients vs. 9/312 controls; odds ratio 3.9, 95% CI 1.8 to 8.3). In bivariate logistic regression, antihistamine use remained a risk factor when adjusted for systemic steroid use (odds ratio 3.2, 95% CI 1.5 to 7.0; P = 0.004) and when adjusted for tobacco use (odds ratio 3.5, 95% CI 1.6 to 7.9; P = 0.002). However, there was no association between antihistamine and CSCR risk in stepwise multivariable modeling (odds ratio 2.5, 95% CI 0.9 to 7.1; P = 0.0855) (24). Allergic respiratory disease was also associated with CSCR (odds ratio 2.5, 95% CI 1.2 to 5.1).

In the prospective matched study, several exposures were more common in CSCR than controls: systemic corticosteroids 14.1% vs 2.6% (P = 0.0173), recent psychological stress or life events 25.6% vs 12.8% (P = 0.0422), smoking (odds ratio 3.19, P<0.05), and antihistaminic use (odds ratio 4.104, P = 0.0466). Alcohol was not significantly different between CSCR and control cases after excluding combined alcohol and smoking (odds ratio 2.60, P = 0.4423). These data describe exposure frequency at diagnosis and do not evaluate the efficacy or safety of antihistamines as CSCR treatment (25).

### 3.4 Visual and anatomical outcomes

In the prospective matched study, 89.7% of CSCR cases achieved good visual recovery at 1 year, defined as best-corrected visual acuity 6/12 or better (25). None of the examined risk factors showed a statistically significant relationship with poor visual recovery at one year. In the management series, most eyes improved anatomically during observation or after focal laser when indicated; the brief antihistamine trial did not significantly change the recovery trajectory (see **Table 1**).

### 3.5 Case presentations: bilateral CSCR flare following discontinuation of antihistamine/mast-cell stabilizer therapy

#### 3.5.1 Case 1

A 63-year-old man with hypertension and hypercholesterolemia, but no history of allergies or exogenous steroid exposure, presented in March 2023 with chronic CSCR affecting both eyes. Multimodal imaging demonstrated features of pachychoroid, diffuse retinal pigment epithelium (RPE) mottling, outer retinal atrophy, and episodic intraretinal and subretinal fluid consistent with chronic CSCR.

The patient was managed with oral loratadine 10 mg twice daily, a selective histamine H1-receptor antagonist that also reduces mast-cell degranulation and histamine-induced choroidal vascular permeability, in combination with topical difluprednate (Durezol), titrated up to four times daily during active phases. This regimen produced anatomic improvement of cystoid macular edema (CME) and subretinal fluid (SRF). After several months of stability, the patient discontinued loratadine for approximately 5–6 weeks in mid-2025 while continuing Durezol once daily. In July 2025, he experienced a sudden bilateral flare characterized by a severe recurrence of CME and SRF in the right eye and new peripapillary and subfoveal SRF in the left eye (visual acuity 20/150 OD, 20/25 OS). The recurrence occurred despite ongoing corticosteroid therapy, and fluorescein and indocyanine-green angiography excluded macular neovascularization (MNV) as a cause of exudation. Loratadine was promptly re-initiated at 10 mg twice daily, and Durezol was intensified to three to four times daily in both eyes.

By September 2025, OCT demonstrated complete resolution of SRF in both eyes and marked reduction of CME in the right eye. Angiography showed only chronic RPE stippling without active leakage, and photodynamic therapy was deferred. The temporal association between loratadine withdrawal and subsequent disease recurrence, followed by anatomical improvement after loratadine re-initiation, is hypothesis-generating; however, interpretation is limited by the simultaneous intensification of difluprednate from once daily to three to four times daily. Therefore, the relative contributions of loratadine re-initiation and corticosteroid intensification to the observed anatomical improvement cannot be determined. This case illustrates a temporal association between re-initiation of loratadine and subsequent anatomical improvement during concurrent corticosteroid intensification, but does not necessarily establish a therapeutic effect of H1-receptor blockade or a synergistic effect with corticosteroids. During periods of corticosteroid escalation, transient intraocular pressure elevations were effectively managed with topical glaucoma medications.

OCT images of the right eye and left eye from all three visits are presented in **Figures 1** and **2**, respectively.

**Figure 1:**
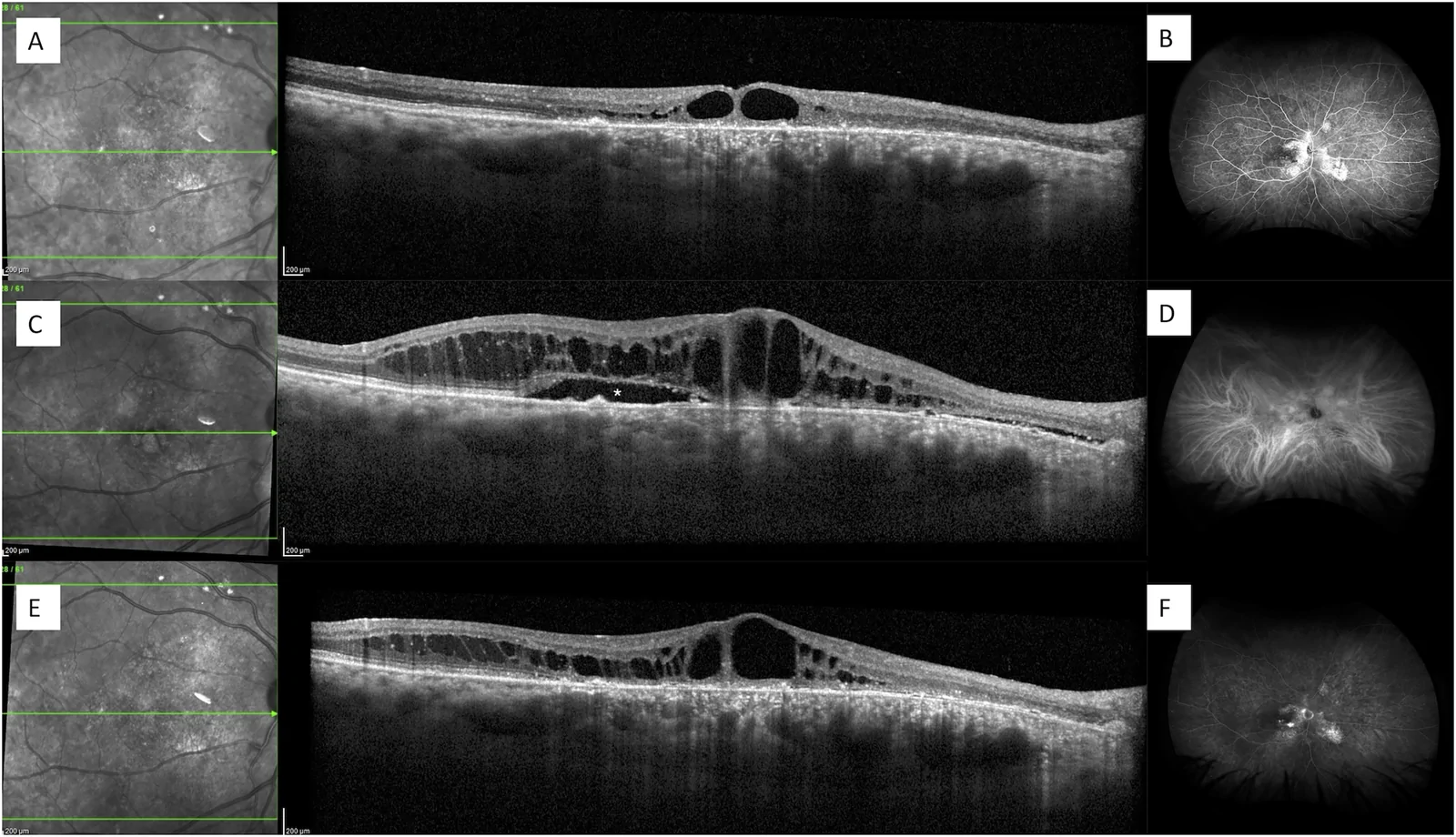
OCT/FAF/FA/ICG OD; Stable baseline (on loratadine BID): Demonstrates fluid control under combined loratadine + Durezol therapy **(A, B)**. After loratadine discontinuation: Shows recurrence, severe CME/SRF OD while on Durezol once daily; MNV excluded by FA/ICG **(C, D)**. After loratadine re-initiation: Demonstrates SRF resolution and CME improvement following re-start of loratadine BID + Durezol TID–QID **(E, F)**.

**Figure 2:**
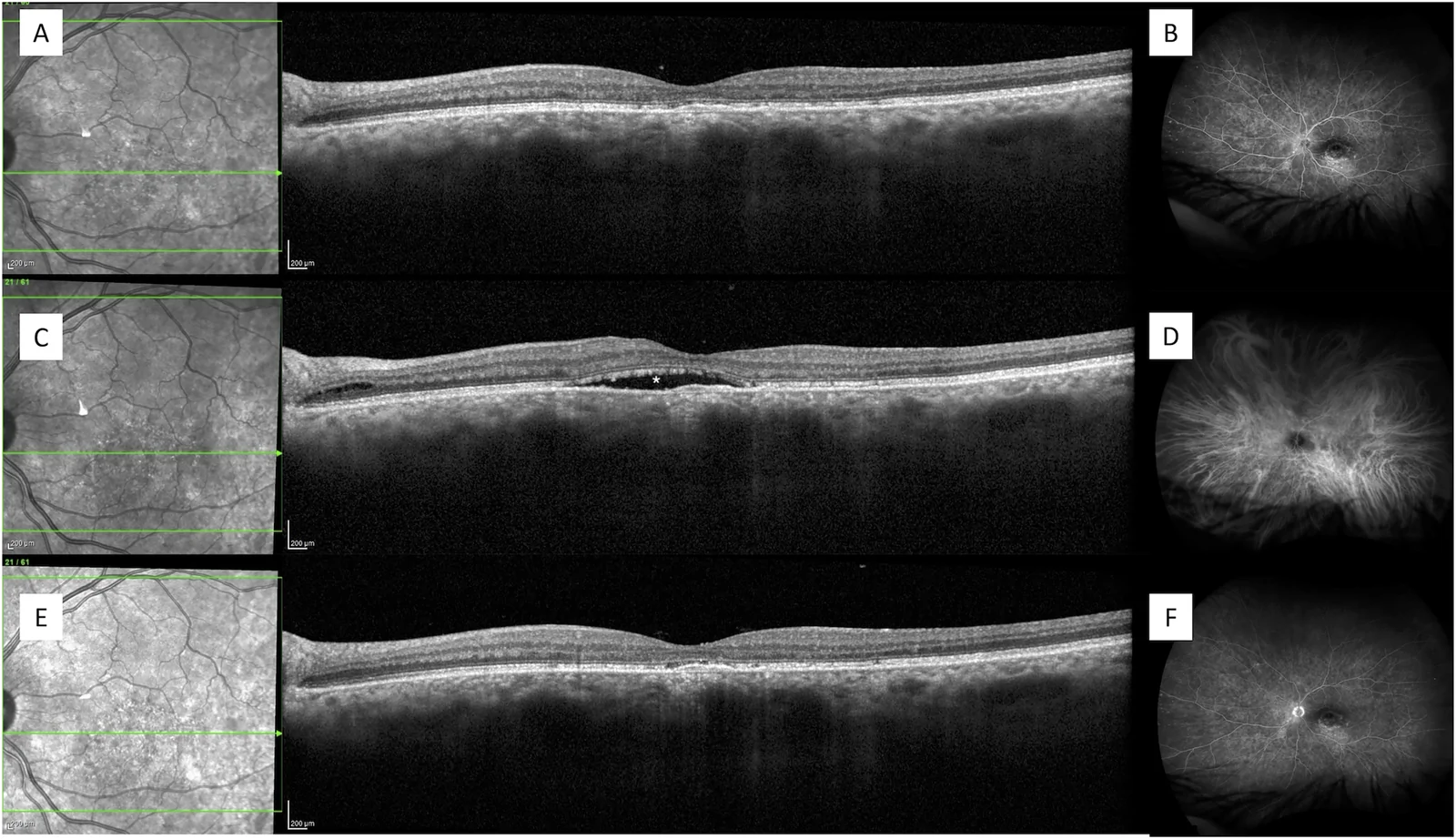
OCT/FAF/FA/ICG OS; Stable baseline (on loratadine BID): Demonstrates fluid control under combined loratadine + Durezol therapy **(A, B)**. After loratadine discontinuation: Shows recurrence, subfoveal SRF OS while on Durezol once daily; MNV excluded by FA/ICG **(C, D)**. After loratadine re-initiation: Demonstrates SRF resolution and CME improvement following re-start of loratadine BID + Durezol TID–QID **(E, F)**.

#### 3.5.2 Case 2

A 39-year-old man with bilateral chronic CSCR since 2017 had recurrent SRF despite PDT (2018, 2020; additional bilateral PDT September 2024) and trials of loratadine, topical steroids limited by steroid-responsive OHT/PSC, and faricimab OD ×3 by 3/2025 in monthly intervals was associated with only a modest, transient effect. In March 2025, Cromolyn sodium 200 mg PO QID was initiated in addition to loratadine BID and nightly montelukast that the patient was already taking. By April 2025, OCT demonstrated a marked bilateral reduction in SRF and CMT with residual shallow detachments. Following dose interruptions and later switch attempts to inhaled cromolyn for bioavailability, control became mixed, with OS/OD SRF fluctuations and renewed consideration of PDT (June-July 2025). Throughout, OCT-angiography (OCT-A) repeatedly excluded MNV, and no heme was observed. The temporal association, robust anatomic improvement shortly after cromolyn introduction, then loss of stability with cromolyn interruption, supports a possible mast-cell–stabilizing effect in this case of treatment-refractory CSCR (**Figure 3**).

**Figure 3:**
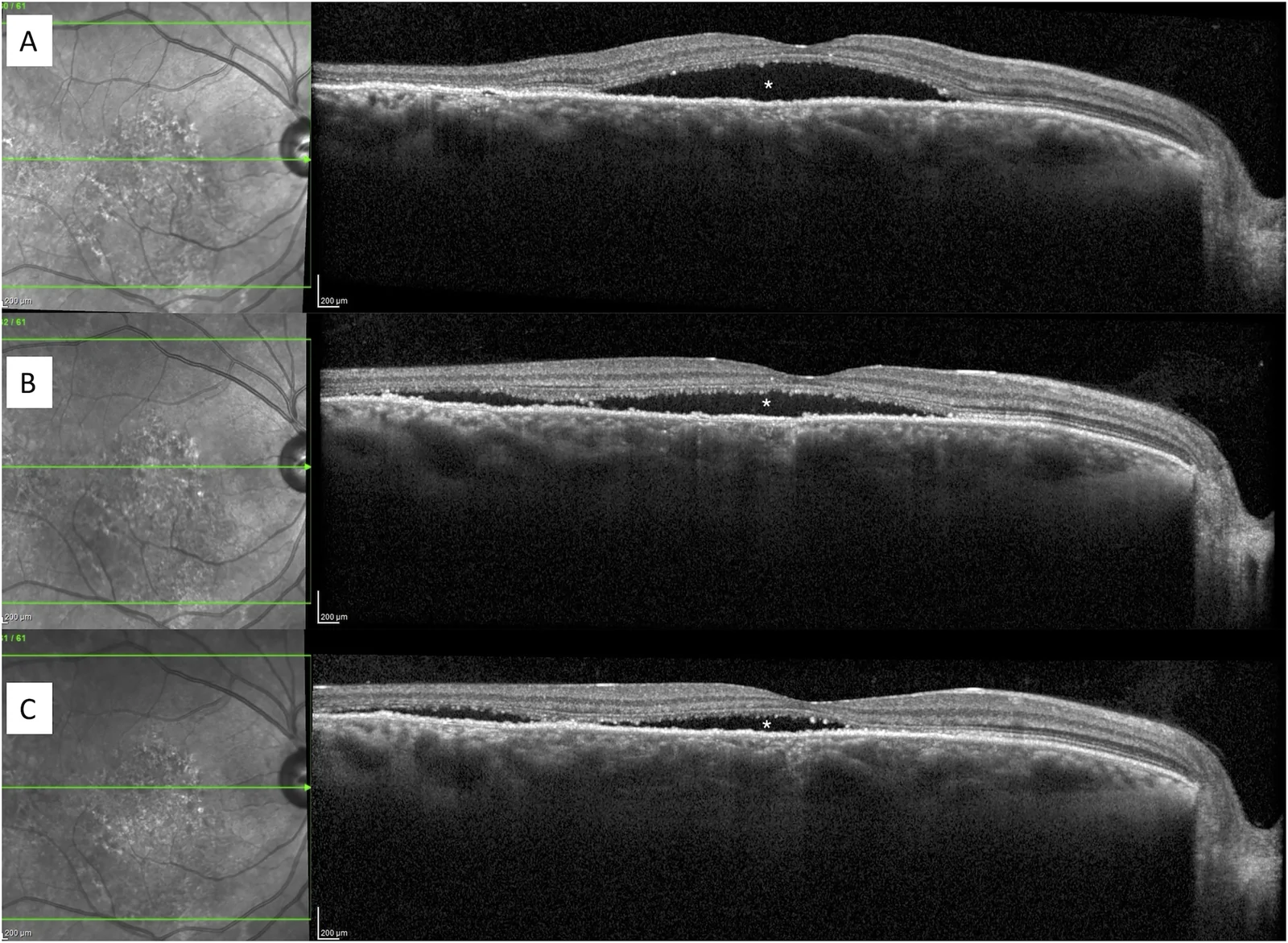
Worsening SRF OU on intensified antihistamine **(A)**. Cromolyn PO QID initiated (with loratadine + montelukast) **(B)**. Best response on cromolyn: major SRF/CMT reduction OU **(C)**.

**Table 2** summarizes the details for both cases.

**Table 2:** Summary of illustrative case reports demonstrating mast cell-targeted therapy in chronic CSCR.

Parameter	Case 1	Case 2
Patient	63-year-old man	39-year-old man
Comorbidities	Hypertension, hypercholesterolemia; no allergies or exogenous steroid exposure	Steroid-responsive ocular hypertension and posterior subcapsular cataract
CSCR phenotype	Chronic bilateral CSCR with pachychoroid features, diffuse RPE mottling, outer retinal atrophy, episodic intraretinal and subretinal fluid	Chronic bilateral CSCR, multi-recurrent, treatment-refractory
Prior treatments	Loratadine 10 mg BID + topical difluprednate (Durezol) titrated up to QID during active phases	PDT (2018, 2020, bilateral Sept 2024); loratadine; topical steroids (limited by OHT/PSC); faricimab OD x3 (modest transient effect)
Mast cell-targeted therapy	Loratadine 10 mg BID (selective H1-receptor antagonist with mast cell-stabilizing properties)	Cromolyn sodium 200 mg PO QID + loratadine BID + montelukast nightly
BCVA (OD;OS)	3/12/2025 (20/150; 20/25); 7/18/2025 (20/150; 20/25); 9/25/2025 (20/150; 20/25); 1/7/2026 (20/150; 20/30)	OS only, 3/21/2025 (20/20); 5/22/2025 (20/20)
SRF (μm) (OD/OS)	7/18/2025 (133/81)	OS only, 3/21/2025 (147); 5/22/2025 (53)
CMT (μm) (OD/OS)	3/12/2025 (256/207); 7/18/2025 (590/265); 9/25/2025 (470/211); 1/7/2026 (395/212)	OS only, 3/21/2025 (316); 5/22/2025 (302)
Key Temporal event	Discontinuation of loratadine for 5–6 weeks (mid-2025) while continuing Durezol QD	Cromolyn initiation March 2025; dose interruptions and switch to inhaled cromolyn for bioavailability
Clinical response	Severe bilateral flare (July 2025): CME/SRF recurrence OD (VA 20/150), new peripapillary and subfoveal SRF OS (VA 20/25). MNV excluded by FA/ICG.	Marked bilateral reduction in SRF and CMT by April 2025 (4 weeks after cromolyn initiation); OS/OD SRF fluctuations with dose interruption
Outcome	Complete SRF resolution OU and marked CME reduction OD by September 2025 after loratadine reinitiation + Durezol TID-QID. PDT deferred.	Loss of stability with cromolyn interruption; renewed consideration of PDT (June-July 2025). OCT-A excluded MNV throughout.
Key finding	Temporal association between loratadine withdrawal and re-challenge supports mast cell-mediated choroidal hyperpermeability; corticosteroid monotherapy was insufficient to maintain remission	Robust anatomic response to cromolyn with loss of control upon interruption supports clinically meaningful mast cell-stabilizing effect in treatment-refractory CSCR

CME, cystoid macular edema; CMT, central macular thickness; CSCR, central serous chorioretinopathy; FA, fluorescein angiography; H1R, histamine H1 receptor; ICG, indocyanine green angiography; MNV, macular neovascularization; OCT-A, optical coherence tomography angiography; OHT, ocular hypertension; PDT, photodynamic therapy; PSC, posterior subcapsular cataract; RPE, retinal pigment epithelium; SRF, subretinal fluid; VA, visual acuity.

## 4 Discussion

This review examined the association between antihistamine use and CSCR, a complex chorioretinal disorder. Synthesizing evidence from three independent observational studies and two illustrative case reports, our findings support a biologically plausible link between mast cell-related pathways and CSCR pathophysiology and management. While causality cannot be inferred, the convergence of epidemiologic, clinical, and mechanistic data warrants further investigation.

Two case-control studies consistently reported a higher frequency of self-reported antihistamine use among patients with CSCR compared with controls (24, 25). In a prospective matched study, antihistamine exposure was associated with substantially increased odds of CSCR (OR 4.104, *P* = 0.0466), whereas a larger multicenter analysis demonstrated a strong univariable association (OR 3.9) that attenuated but remained near statistical significance after multivariable adjustment (OR 2.5, *P* = 0.0855) (24, 25). Importantly, these findings reflect antihistamine exposure at or near the time of CSCR diagnosis and do not establish a causal relationship. Rather, the observed association may reflect the presence, or treatment, of an underlying inflammatory or allergic state in individuals predisposed to pachychoroid pathology. More likely, antihistamine use may mark underlying allergic comorbidity in individuals predisposed to pachychoroid disease, rather than represent an independent risk factor. Importantly, an association between antihistamine exposure and CSCR should therefore be distinguished from evidence of histamine pathway activation and, separately, from evidence that antihistamines are therapeutically effective once CSCR has developed. Thus, whether antihistamines identify an at-risk population is distinct from whether they have therapeutic value in CSCR.

Evidence supporting antihistamines as a direct therapeutic intervention for CSCR remains limited and inconclusive. A single uncontrolled management series involving 40 patients demonstrated minimal benefit from a brief four-day course of antihistamines, with 29 of 30 patients showing no rapid anatomical or visual improvement (26). The high rate of spontaneous resolution observed during the subsequent 5-10-week placebo phase (21 of 33 patients) suggests that the natural history of acute CSCR largely accounted for recovery, likely obscuring any modest treatment effect. Notably, no studies have evaluated sustained antihistamine therapy or mast cell stabilizers in either acute or chronic CSCR, nor have dose-response relationships been examined. Thus, the available clinical literature does not currently provide evidence sufficient to support antihistamines or mast-cell stabilizers as established treatments for CSCR.

In contrast, our two case reports introduce a potentially important and previously underexplored therapeutic dimension involving mast cell modulation in chronic, treatment-refractory CSCR. In Case 1, discontinuation of oral loratadine, an H1-receptor antagonist with mast cell-stabilizing properties, was temporally associated with a sudden, severe bilateral CSCR recurrence, despite continued low-dose topical corticosteroid therapy (difluprednate). Anatomical improvement subsequently occurred after loratadine was re-initiated; however, difluprednate was simultaneously intensified to three to four times daily. Therefore, the observed improvement cannot be confidently attributed to either loratadine or mast-cell modulation alone. The temporal pattern is hypothesis-generating and may be consistent with potential contribution of histamine-related pathways, but the concurrent corticosteroid escalation represents an important confounding factor. Accordingly, this case does not establish an additive or synergistic therapeutic effect between H1-receptor blockade and corticosteroids. Case 2 provides an additional hypothesis-generating temporal association between initiation and interruption of cromolyn therapy and changes in subretinal fluid. However, the patient was receiving multiple concomitant therapies and underwent treatment modifications during follow-up, limiting attribution of the observed anatomical changes to cromolyn alone. These findings therefore cannot establish the efficacy or clinical relevance of mast-cell stabilization in chronic CSCR.

Mast cells are key effectors of innate immunity, and their abundance within the uveal tract and choroid suggests a fundamental role in ocular immune homeostasis (27). Beyond classical triggers such as pathogens and toxins, mast cells respond to endogenous stimuli present within the choroidal milieu. In geographic atrophy, for example, advanced glycation end products, complement fragments (C3a and C5a), and C-reactive protein have all been identified as mast cell activators (28–31).

CSCR is increasingly recognized as part of the pachychoroid disease spectrum, characterized by choroidal thickening, vascular hyperpermeability, and dysregulated blood flow. The pathogenesis is multifactorial, with glucocorticoid exposure representing the most established risk factor through overactivation of mineralocorticoid receptors (MR) in the retina and choroid. Recent work has identified the choroidal nervous system (ChNS) as a key regulator of choroidal circulation, with dense innervation and neuroimmune interfaces involving calcitonin gene-related peptide (CGRP)-containing vesicles closely opposed to choroidal macrophages. Transgenic mice overexpressing human MR exhibit a pachychoroid-like phenotype with choroidal neuropathy, and transcriptomic profiling reveals dysregulation of pathways related to vascular development, hypoxia, and inflammation. These findings suggest that MR-induced choroidal neuropathy may link corticosteroid exposure, systemic dysautonomia, and pachychoroid-driven disorders such as CSCR (32).

Recent human proteomic and histologic studies provide direct evidence that mast cell infiltration and degranulation within the choroid constitute an early, genetically driven event preceding overt retinal disease (33). Quantitative mass spectrometry of submacular choroidal tissue from donors without clinical AMD but carrying high-risk haplotypes at *CFH* (Y402H) or *HTRA1/ARMS2* revealed significantly elevated levels of mast cell-specific proteases, including tryptase, chymase, and carboxypeptidase A3, compared with genetically low-risk controls (33). These molecular alterations were present in the absence of clinical disease, indicating that mast cell activation may occur upstream of overt pathology (33). Histologic analyses further demonstrated increased mast cell density within the inner choroid, particularly tryptase-positive/chymase-negative cells, alongside collagen denaturation and extracellular matrix remodeling, supporting a direct role for mast cell degranulation in choroidal vascular instability and susceptibility to serous retinal detachment (33).

Genetic association studies further reinforce the importance of complement-mediated choroidal vascular dysfunction in CSCR (34). A large three-cohort analysis involving 1,176 patients with CSCR and 526,787 controls identified five CSCR risk loci, including replication of loci near *CFH* and *GATA5* and identification of novel loci near *CD34/46*, *NOTCH4*, and *PREX1* (34). Notably, *CFH* and *NOTCH4* were also associated with age-related macular degeneration, but in the opposite direction, highlighting divergent phenotypic outcomes arising from shared complement-related genetic architecture (34). Prioritized genes showed increased expression in cultured choroidal endothelial cells and were significantly enriched in choroidal vascular endothelial cells on single-cell RNA sequencing, underscoring the central role of choroidal vascular regulation (34). Moreover, a polygenic risk score for AMD was associated with reduced CSCR risk, an effect largely mediated by complement-related loci (34). Together, these data link genetic dysregulation of complement activity to mast cell activation and choroidal hyperpermeability in CSCR.

CSCR and AMD share genetic associations at the CFH locus but with opposite direction of effect: risk alleles for AMD are protective for CSCR, implying distinct endpoints of complement dysregulation (35). In AMD, excessive complement activation drives drusen formation and neovascularization, whereas in CSCR, reduced complement regulation may predispose to choroidal hyperpermeability through mast cell priming. Imaging distinguishes pachychoroid diseases from drusen-driven AMD by their thickened choroid with dilated pachyvessels and vascular hyperpermeability on indocyanine green angiography (35). Subfoveal choroidal thickness in CSCR significantly exceeds that of typical neovascular AMD. This convergence of complement genetics and choroidal vascular architecture suggests that CFH variants facilitating chronic complement activation prime resident mast cells, perpetuating the hyperpermeability characteristic of pachychoroid spectrum diseases.

A variety of physiologic and pathologic stimuli, including stress, temperature changes, mechanical vibration, medications, complement fragments, peptides, and acetylcholine, can trigger mast cell activation (35, 36). Upon degranulation, mast cells release vasoactive mediators such as histamine, promoting neutrophil recruitment and local inflammation (37). In the eye, this process may induce choroidal vasodilation, increased vascular permeability, and subsequent serous retinal detachments (7). These mechanisms provide a plausible explanation for the vascular hyperpermeability observed in CSCR and identify mast cells as a potential therapeutic target.

The immunological basis for antihistamine efficacy in CSCR involves histamine dual action on choroidal vasculature and RPE. Histamine induces vascular hyperpermeability via H1 receptor-mediated vasodilation and disruption of vascular endothelial cadherin (VE-cadherin) at endothelial junctions, both abolished by H1 antagonism but not H2 blockade (38). In human RPE cells, H1 receptors are the predominant histamine receptor subtype; stimulation triggers intracellular calcium mobilization that is completely suppressed by H1 antagonists (39). This calcium signaling may impair RPE tight junction integrity and ion transport, compromising the outer blood-retinal barrier. Thus, H1-selective antihistamines may simultaneously reduce choroidal hyperpermeability and restore RPE barrier function, while mast cell stabilizers act upstream by preventing degranulation and the release of histamine, tryptase, and pro-inflammatory cytokines.

Consistent with these observations, a reported case of advanced systemic mastocytosis demonstrated acute central vision loss attributed to presumed ocular mast cell infiltration (40). Additional studies have shown increased choroidal mast cell density and enhanced degranulation in geographic atrophy. Experimental work by Bousquet and colleagues demonstrated that mast cell degranulation induces choroidal thickening and serous detachments, whereas inhibition of this process prevents such changes (7).

Basic science data further support the biological plausibility of antihistamine and mast cell-stabilizing therapies in CSCR. *In vivo* human studies demonstrate that histamine increases subfoveal choroidal blood flow and retinal vessel diameter, effects attenuated by H1-receptor blockade with diphenhydramine but not by H2-receptor blockade with cimetidine (41). Experimental mast cell degranulation induces choroidal vasodilation, increased permeability, and serous retinal detachment, while pretreatment with cromolyn mitigates these effects (7). Mast cell proteases, such as tryptase, can disrupt outer retinal barrier integrity, linking mast cell activation to subretinal fluid accumulation in pachychoroid disorders (42). A summary of proposed mechanistic pathways is provided in **Table 3**.

**Table 3:** Proposed mechanistic pathway: mast cell/histamine Axis in CSCR pathophysiology and therapeutic targets.

Pathway step	Mechanism	Supporting evidence	Therapeutic target
1. Mast cell priming and activation	IgE cross-linking at FcϵRI; complement C3a/C5a via alternative pathway; neuropeptide stimulation (CGRP); corticosteroid-induced choroidal milieu changes	CFH risk variants in CSCR (12); elevated mast cell proteases in high-risk genotypes (33); CSCR in atopic patients (11)	Mast cell stabilizers (cromolyn, nedocromil, ketotifen); complement inhibitors
2. Mast cell degranulation	Release of preformed mediators: histamine, tryptase, chymase, TNF-α, leukotrienes, proteoglycans	Subconjunctival compound 48/80 causes choroidal vasodilation and serous detachments in mice; reduced by cromoglycate (7)	Mast cell stabilizers (cromolyn sodium, ketotifen)
3. H1R activation on choroidal vasculature	NO-mediated vasodilation; VE-cadherin disruption at endothelial junctions; increased blood flow velocity/volume; increased hydrostatic pressure	Histamine increases choroidal blood flow via H1R, blocked by diphenhydramine not cimetidine (9); H1R mediates vascular hyperpermeability via VE-cadherin disruption (38)	H1 antihistamines (loratadine, cetirizine, fexofenadine, diphenhydramine)
4. H1R activation on RPE	Intracellular Ca2+ mobilization via H1R; tight junction disruption; impaired ion/fluid transport; circadian clock phase shifts affecting RPE physiology	H1R is predominant histamine receptor in human RPE cells; histamine induces concentration-dependent Ca2+ rises, completely blocked by H1R antagonists (39)	H1 antihistamines; RPE-targeted therapies
5. Choroidal vascular hyperpermeability and SRF	Plasma extravasation into sub-RPE space; increased choroidal hydrostatic pressure overwhelms RPE pump; subretinal fluid accumulation	IV histamine increases subfoveal choroidal blood flow in humans (8, 41); serous retinal detachments in mast cell degranulation models (7)	H1 antihistamines + mast cell stabilizers; leukotriene inhibitors (montelukast)
6. Feed-forward amplification	Histamine amplifies mast cell activity via H1/H4 autocrine receptors; TNF-α recruits neutrophils and macrophages; chronic inflammatory cycle perpetuates hyperpermeability	Mast cell autocrine histamine loop (10); TNF-α-dependent neutrophil recruitment (37); tryptase disrupts outer retinal barrier (42)	Combined H1 blockade + mast cell stabilization + leukotriene pathway inhibition

Ca2+, calcium; CGRP, calcitonin gene-related peptide; CME, cystoid macular edema; CMT, central macular thickness; CSCR, central serous chorioretinopathy; FA, fluorescein angiography; H1R, histamine H1 receptor; ICG, indocyanine green angiography; IgE, immunoglobulin E; MNV, macular neovascularization; NO, nitric oxide; OCT-A, optical coherence tomography angiography; OHT, ocular hypertension; PDT, photodynamic therapy; PSC, posterior subcapsular cataract; RPE, retinal pigment epithelium; SRF, subretinal fluid; TNF-α, tumor necrosis factor alpha; VA, visual acuity; VE-cadherin, vascular endothelial cadherin.

While systemic corticosteroid exposure is a well-established risk factor for the development or exacerbation of CSCR, the role of topical ophthalmic corticosteroids appears more nuanced and context-dependent. Population-based evidence has demonstrated a significant association between recent or persistent use of topical ophthalmic corticosteroids and increased CSCR risk, likely due to local diffusion to posterior segment tissues or minor systemic absorption. Patients requiring such drops should therefore be monitored closely for signs of CSCR (43).

However, recent studies suggest potential therapeutic benefits of topical corticosteroids in select CSCR cases or related pachychoroid spectrum disorders, particularly those with peripapillary involvement or chronic/recurrent features. This may reflect differences in dosing, duration, underlying pathophysiology (e.g., inflammatory components or local steroid dysregulation), or secondary effects such as mild intraocular pressure elevation potentially tamponading choroidal leakage (44). Zola et al. found that in eyes with long-lasting complex inactive CSCR, aqueous humor showed lower active glucocorticoid (cortisol) levels and a shift favoring active mineralocorticoids. This local dysregulation, independent of systemic levels, led the authors to trial combined approaches, including topical glucocorticoids in refractory cases, with favorable outcomes. These findings highlight a potential deficiency in chronic CSCR that topical supplementation might address (45). In case 1, topical corticosteroid exposure was not constant; difluprednate was being administered once daily prior to disease recurrence and was subsequently intensified to three to four times daily when loratadine was reinitiated. This concurrent change in corticosteroid exposure represents an important potential confounder and limits attribution of the subsequent anatomical improvement to loratadine. Therefore, the clinical course should not be interpreted as evidence of an isolated antihistamine effect.

This study has several limitations. The quantitative synthesis is constrained by the small number and heterogeneity of included studies. Therapeutic evidence is limited to a single, small, uncontrolled series with brief antihistamine exposure, precluding meaningful assessment of long-term efficacy or safety. Medication exposure data are subject to recall bias, and the observed association between antihistamine use and CSCR may reflect residual confounding, such as severity of underlying allergic disease. Finally, although our case reports demonstrate compelling temporal associations, they are anecdotal and lack generalizability, particularly with respect to optimal dosing, duration, and patient selection for mast cell-targeted therapies.

## 5 Conclusion

Current evidence does not support brief antihistamine therapy as primary treatment for acute CSCR, given its limited benefit beyond the high rate of spontaneous resolution. Associations between antihistamine exposure, allergic comorbidity, and CSCR risk should be distinguished from evidence of histamine pathway activation or therapeutic efficacy. The two illustrative cases provide hypothesis-generating temporal observations regarding anti-allergic therapy and CSCR activity, but their interpretation is limited by small sample size and concurrent treatment changes, particularly corticosteroid intensification in case 1.

Future randomized controlled trials should evaluate sustained regimens of selective H1 antagonists and/or mast cell stabilizers, either as monotherapy or adjunctive treatment in chronic CSCR. Whether mast-cell-directed therapies have any additive or synergistic effects when combined with corticosteroids remains unknown and should be evaluated prospectively. CSCR is a heterogenous disorder in which inflammatory and immune-related pathways may contribute in some phenotypes; mast-cell and histamine-related pathways therefore represent a biologically plausible but unproven area for further investigation.

## Data Availability

The raw data supporting the conclusions of this article will be made available by the authors, without undue reservation.
