# Supplementary material for: Case Report: Histamine-mediated inflammation and mast cell modulation in central serous chorioretinopathy: a two case illustrations
Source: Front Immunol. 2026 Oct 7;17:1799560. doi: 10.3389/fimmu.2026.1799560 (PMC13683630; doi:10.3389/fimmu.2026.1799560)
Supplement: Supplementary file 1 [file Table1.docx]

**Supplementary Materials**

**Table S1.** Search Strategies

| Database (11/02/2025) | Code Line | Search Strategy | Number of Results |
| --- | --- | --- | --- |
| **Embase** | 1 | exp central serous chorioretinopathy/ | 208 |
|  | 2 | (central serous chorioretinopath* or central serous retinopath* or ((central adj2 serous) adj2 (retinopath* or chorioretinopath*)) or CSCR or CSR or CSC).ti,ab,kw. |  |
|  | 3 | (pachychoroid* or pachyvessel* or "pachychoroid spectrum" or "pachychoroid pigment epitheliopathy" or PPE or "peripapillary pachychoroid syndrome" or (peripapillary adj2 pachychoroid*) or "pachychoroid neovasculopathy" or PNV).ti,ab,kw. |  |
|  | 4 | exp polypoidal choroidal vasculopathy/ |  |
|  | 5 | ("polypoidal choroidal vasculopathy" or PCV).ti,ab,kw. |  |
|  | 6 | 4 or 5 |  |
|  | 7 | (macular neovascular* or MNV or CNV or (choroid* adj2 neovascular*) or (choroidal adj2 neovascular*) or ("type 1" adj2 neovascular*)).ti,ab,kw. |  |
|  | 8 | 6 not 7 |  |
|  | 9 | 1 or 2 or 3 or 8 |  |
|  | 10 | exp mast cell/ |  |
|  | 11 | exp histamine/ or exp histamine receptor antagonist/ or exp histamine H1 receptor antagonist/ or exp histamine H2 receptor antagonist/ |  |
|  | 12 | (mast cell* or mast-cell* or (mast adj1 cell* adj1 stabiliz*) or histamin* or antihistamin* or (H1 adj2 antagon*) or (H2 adj2 antagon*)).ti,ab,kw. |  |
|  | 13 | (cromolyn or cromoglycate or cromoglicate or "sodium cromoglicate" or "cromoglicic acid" or lodoxamide or ketotifen or nedocromil or olopatadine or bepotastine or epinastine or azelastine).ti,ab,kw. |  |
|  | 14 | exp cromoglicic acid/ or exp lodoxamide/ or exp ketotifen/ or exp nedocromil/ or exp olopatadine/ or exp azelastine/ |  |
|  | 15 | exp leukotriene receptor antagonist/ or exp montelukast/ or exp zafirlukast/ or exp pranlukast/ |  |
|  | 16 | (montelukast or zafirlukast or pranlukast).ti,ab,kw. |  |
|  | 17 | 10 or 11 or 12 or 13 or 14 or 15 or 16 |  |
|  | 18 | 9 and 17 |  |
| Medline | 1 | 1. exp Chorioretinopathy, Serous Central/ | 101 |
|  | 2 | (central serous chorioretinopath* or central serous retinopath* or ((central adj2 serous) adj2 (retinopath* or chorioretinopath*)) or CSCR or CSR or CSC).ti,ab,kf. |  |
|  | 3 | (pachychoroid* or pachyvessel* or "pachychoroid spectrum" or "pachychoroid pigment epitheliopathy" or PPE or "peripapillary pachychoroid syndrome" or (peripapillary adj2 pachychoroid*) or "pachychoroid neovasculopathy" or PNV).ti,ab,kf. |  |
|  | 4 | ("polypoidal choroidal vasculopathy" or PCV).ti,ab,kf. |  |
|  | 5 | ((macular adj1 neovascular*) or MNV or CNV or (choroid* adj2 neovascular*) or (choroidal adj2 neovascular*) or ("type 1" adj2 neovascular*)).ti,ab,kf. |  |
|  | 6 | 4 not 5 |  |
|  | 7 | 1 or 2 or 3 or 6 |  |
|  | 8 | exp Mast Cells/ |  |
|  | 9 | exp Histamine/ or exp Histamine Antagonists/ or exp Histamine H1 Antagonists/ or exp Histamine H2 Antagonists/ |  |
|  | 10 | (mast cell* or mast-cell* or (mast adj1 cell* adj1 stabiliz*) or histamin* or antihistamin* or (H1 adj2 antagon*) or (H2 adj2 antagon*)).ti,ab,kf. |  |
|  | 11 | (cromolyn or "cromolyn sodium" or cromoglycate or cromoglicate or "sodium cromoglicate" or "cromoglicic acid" or lodoxamide or ketotifen or nedocromil or olopatadine or bepotastine or epinastine or azelastine).ti,ab,kf. |  |
|  | 12 | Cromolyn Sodium/ or Ketotifen/ or Nedocromil/ or Azelastine/ |  |
|  | 13 | exp Leukotriene Antagonists/ or Montelukast/ or Zafirlukast/ or Pranlukast/ |  |
|  | 14 | (montelukast or zafirlukast or pranlukast).ti,ab,kf. |  |
|  | 15 | 8 or 9 or 10 or 11 or 12 or 13 or 14 |  |
|  | 16 | 7 and 15 |  |
| Cochrane | 1 | #1 (central serous chorioretinopath* OR central serous retinopath* OR    ((central NEAR/2 serous) NEAR/2 (retinopath* OR chorioretinopath*)) OR    CSCR OR CSR OR CSC):ti,ab,kw | 35 |
|  | 2 | (pachychoroid* OR pachyvessel* OR "pachychoroid spectrum" OR    "pachychoroid pigment epitheliopathy" OR PPE OR    "peripapillary pachychoroid syndrome" OR (peripapillary NEAR/2 pachychoroid*) OR    "pachychoroid neovasculopathy" OR PNV):ti,ab,kw |  |
|  | 3 | ("polypoidal choroidal vasculopathy" OR PCV):ti,ab,kw |  |
|  | 4 | ((macular NEAR/1 neovascular*) OR MNV OR CNV OR    (choroid* NEAR/2 neovascular*) OR (choroidal NEAR/2 neovascular*) OR    ("type 1" NEAR/2 neovascular*)):ti,ab,kw |  |
|  | 5 | #3 NOT #4 |  |
|  | 6 | #1 OR #2 OR #5 |  |
|  | 7 | (mast cell* OR mast-cell* OR (mast NEAR/1 cell* NEAR/1 stabiliz*) OR    histamin* OR antihistamin* OR ("H1" NEAR/2 antagon*) OR ("H2" NEAR/2 antagon*)):ti,ab,kw |  |
|  | 8 | (cromolyn OR "cromolyn sodium" OR cromoglycate OR cromoglicate OR    "sodium cromoglicate" OR "cromoglicic acid" OR lodoxamide OR ketotifen OR    nedocromil OR olopatadine OR bepotastine OR epinastine OR azelastine):ti,ab,kw |  |
|  | 9 | (montelukast OR zafirlukast OR pranlukast):ti,ab,kw |  |
|  | 10 | #7 OR #8 OR #9 |  |
|  | 11 | #6 AND #10 |  |

**Table S2.** PRISMA flow diagram

**Identification of studies via databases and registers**

Records identified from:

Embase (n = 208)

Medline (n = 101)

Cochrane (n = 42)

Records removed *before screening*:

Duplicate records removed (n = 35)

**Identification**

Records screened

(n = 316)

Records excluded

(n = 308)

Reports sought for retrieval

(n = 8)

Reports not retrieved

(n = 0)

**Screening**

Reports excluded:

Conference paper (n = 1)

Case report (n = 2)

Wrong outcome (n = 2)

Reports assessed for eligibility

(n = 8)

Studies included in review

(n = 3)

**Included**

**Table S5.** ROBINS-I risk of bias assessment for Murthy et al. (1983)

| **Domain** | **Judgment** | **Support for judgment** |
| --- | --- | --- |
| Confounding | Serious | No control group for antihistamine arm; no adjustment for confounders |
| Selection of participants | Serious | Convenience sample, single center; allocation to antihistamine group not randomized (30/40 pts) |
| Classification of interventions | Low | Intervention clearly defined (chlorpheniramine 4mg TID x4 days) |
| Deviations from intended interventions | Serious | After 4 days, switched to vitamin C placebo; non-responders got laser. Cannot isolate antihistamine effect from spontaneous resolution |
| Missing data | Moderate | Outcomes reported for all patients but follow-up duration/completeness not fully specified |
| Measurement of outcomes | Serious | Unmasked assessment by treating physician; no independent adjudication |
| Selection of reported result | Moderate | No pre-specified analysis plan; rationale for 4-day treatment duration unclear |
| **Overall** | Serious | No randomization, no controls, unmasked assessment, sequential treatment protocol |

*ROBINS-I = Risk Of Bias In Non-randomised Studies of Interventions.*

**Table S6.** Newcastle-Ottawa Scale assessment for case-control studies

| **NOS Item** | **Haimovici 2004** | **Das 2020** | **Notes** |
| --- | --- | --- | --- |
| **Selection** |  |  |  |
| Case definition adequate | * | * | Both confirmed by clinical exam + angiography/OCT |
| Representativeness of cases | * | * | Consecutive cases; Haimovici multicenter, Das single center |
| Selection of controls | * | * | Hospital/clinic controls |
| Definition of controls | * | * | No history of CSCR |
| **Comparability** |  |  |  |
| Main factor (age/sex) | * | * | Both matched on age and sex |
| Additional factors | * | - | Haimovici: stepwise logistic regression. Das: mostly univariate |
| **Exposure** |  |  |  |
| Ascertainment of exposure | * | * | Structured interview/questionnaire |
| Same method for cases/controls | * | * |  |
| Non-response rate | - | - | Not reported in either study |
| **Total** | **8/9** | **7/9** |  |

** = star awarded; - = not awarded. Maximum score = 9.*

**Table S7.** GRADE certainty of evidence

| **Outcome** | **Risk of bias** | **Inconsistency** | **Indirectness** | **Imprecision** | **Certainty** |
| --- | --- | --- | --- | --- | --- |
| Antihistamine use as risk factor for CSCR (Haimovici 2004; Das 2020) | Serious (-1) | Not serious | Serious (-1)† | Serious (-1)‡ | Very low |
| Antihistamine efficacy in CSCR (Murthy 1983) | Serious (-1) | N/A | Serious (-1)§ | Serious (-1) | Very low |

*Starting certainty is Low for observational studies.*

*† Case-control studies assess antihistamines as a risk factor, not a treatment.*

*‡ Wide CIs; Haimovici antihistamine OR 2.5 (P=0.09), Das OR 4.1 (P=0.047); small exposed subgroups.*

*§ 4-day antihistamine course insufficient to assess long-term efficacy; differen*
